# The Concilium of Information Processing Networks of Chemical Oscillators for Determining Drug Response in Patients With Multiple Myeloma

**DOI:** 10.3389/fchem.2022.901918

**Published:** 2022-07-08

**Authors:** Ashmita Bose, Peter Dittrich, Jerzy Gorecki

**Affiliations:** ^1^ Institute of Physical Chemistry, Polish Academy of Sciences, Warsaw, Poland; ^2^ Department of Mathematics and Computer Science, Friedrich Schiller University Jena, Jena, Germany

**Keywords:** chemical computing, oscillations, Oregonator model, networks, genetic optimization, multiple myeloma, gene expression values

## Abstract

It can be expected that medical treatments in the future will be individually tailored for each patient. Here we present a step towards personally addressed drug therapy. We consider multiple myeloma treatment with drugs: bortezomib and dexamethasone. It has been observed that these drugs are effective for some patients and do not help others. We describe a network of chemical oscillators that can help to differentiate between non-responsive and responsive patients. In our numerical simulations, we consider a network of 3 interacting oscillators described with the Oregonator model. The input information is the gene expression value for one of 15 genes measured for patients with multiple myeloma. The single-gene networks optimized on a training set containing outcomes of 239 therapies, 169 using bortezomib and 70 using dexamethasone, show up to 71% accuracy in differentiating between non-responsive and responsive patients. If the results of single-gene networks are combined into the concilium with the majority voting strategy, then the accuracy of predicting the patient’s response to the therapy increases to ∼ 85%.

## 1 Introduction

The medical therapy of the future will be based on drugs individually selected for patient needs. Individual approach is necessary because it has been observed that one standard treatment does not work for all patients with the same type of cancer (Mulligan et al., 2007). An example is the therapy for patients with multiple myeloma. The drugs as bortezomib or dexamethasone have shown a good anti-myeloma effect, and they has been approved for treatment for patients with multiple myeloma (Field-Smith et al., 2006;^1^). But the literature reports suggest that myeloma consists of many variants with different molecular pathologies (Hideshima et al., 2004; Mulligan et al., 2007). For certain subtypes, the drugs mentioned above can be effective, and for others, they may not. We can hope that using the gene expression profiling one can determine if certain drugs will be effective on a particular patient or not (Lesko and Woodcock, 2004). Considering the challenges one can face with genomic analysis for each patient (Zhan et al., 2006), we present a method that can be used to predict the drug effectiveness if the expression values of selected genes are known.

Our study is based on the results published in (Mulligan et al., 2007), its Supplementary Material (^2^) and the data of clinical tests available on the related web page (^3^). We considered this important medical problem to demonstrate the power of chemical computers operating with nonlinear chemical processes (Adamatzky et al., 2005). There are zillions of chemical computers operating around us because information processing in living organisms is based on chemical reactions. Animals and humans, using their nerve systems and brains, can control complex life processes, create models of the environment they function and even develop self-awareness. It demonstrates that Nature-made chemical computers can perform very complex computational tasks at low energy consumption. However, the development of human-made chemical computers has not shown as spectacular progress as semiconductor microprocessors (Waldrop, 2016). The binary information coding combined with robust logic gates, perfectly suited for electronic computers (Feynman et al., 2000), does not work for chemical computers because the lifetime of reagents is short. Therefore, the bottom-up approach (Feynman et al., 2000) does not help to design efficient chemical information processing devices. There are a few examples of the high computing potential of a chemical medium and its ability for parallel processing, such as the prairie-fire algorithm for labyrinth search (Steinbock et al., 1995) or image processing with a photosensitive variant of oscillatory Belousov-Zhabotinsky reaction (Kuhnert et al., 1989). However, the number of such human-proposed, efficient algorithms is limited. An alternative to the cleverness of a human researcher is the top-down machine design, where the computing medium is optimized to perform a selected task. The design of a chemical McCulloch-Pits neuron (McCulloch and Pitts, 1943) is difficult and requires high precision in setting the medium parameters (Gorecka and Gorecki, 2006). In this respect, networks of interacting chemical oscillators seem to be an interesting candidate for chemical computers. The recent results (Adamatzky et al., 2011; Holley et al., 2011; Szymanski et al., 2011; Adamatzky et al., 2012) demonstrated that networks of chemical oscillators can be easily assembled in experiments and studied for around a day (Muzika and Górecki, 2022). Such networks can be optimized to perform classification tasks (Gizynski et al., 2017) and process information with the best possible use of the chemical medium. The top-down design allows to design oscillator networks that perform functions for which a straightforward algorithm does not exist, as the determination of the cancer type on the basis of medical tests (Gizynski and Gorecki, 2017a).

In this paper, we describe another application of chemical oscillator networks for a medically oriented problem. We present a method that can help to determine the outcome of multiple myeloma therapy with bortezomib or dexamethasone drugs. The method is supposed to determine the response of a patient with a given gene expression profile to the therapy with the drugs mentioned above. The expression values of genes listed in Table 1 were considered. The functions of selected genes can be found in (^4^). For example, the gene RPS7 we consider in the discussed example of a single gene classifier encodes a ribosomal protein that belongs to the S7E family of ribosomal proteins. The gene CXCL5 encodes a protein that is a member of the CXC subfamily of chemokines, which recruit and activate leukocytes. This protein is proposed to bind the G-protein coupled receptor chemokine (C-X-C motif) receptor 2 to recruit neutrophils, promote angiogenesis, and remodel connective tissues. It is believed to play a role in cancer cell proliferation, migration, and invasion. The gene SERP1 is predicted to be involved in the endoplasmic reticulum unfolded protein response and protein glycosylation and act within several processes, including multicellular organism aging, positive regulation of organ growth, and positive regulation of peptide hormone secretion.

**TABLE 1:** Genes considered for determining the drug response and their range.

Gene no. *i*	Gene name	Gene expression value range in the set *R*: *Min*_*k*_(*e*_*i*,*k*_) − *Max*_*k*_(*e*_*i*,*k*_)	Histogram accuracy (%)
1	SERP1	315.8–1879.8	56.9
2	NPM1	1,556.8–9,849.1	56.9
3	PIK3R1	59.8–437.1	57.7
4	APEX1	200.7–1741.2	58.9
5	DAPP1	69.9–564.5	59.4
6	NRAS	38.8–679.2	55.6
7	RRAGC	148.9–679.2	61.1
8	CFLAR	135.7–2000.7	56.9
9	CXCL5	1.09–58.8	57.3
10	IL15	13.3–562.05	58.9
11	NFK*β*2	54.7–2,848.01	56.9
12	COX7C	559.5–5,476.9	61.5
13	RPS7	1,142.48–12,167.3	62.3
14	RPS13	2079.7–23,208.7	60.6
15	UQCRH	370.82–5,554.15	60.2

In our study, we used the gene expression values provided by the authors of reference (Mulligan et al., 2007). Total RNA was isolated using Qiagen RNAeasy kit (^5^), and the expression values were measured by using the microarray technique (^6^). The information on the detailed procedure of myeloma cells enrichment, producing the expression of genes in myeloma cells, and quality control metrics can be found in Document 1 listed at the end of the web page (^7^). The records of the training dataset containing the gene expression values were generated from the GSM files describing the clinical results that can be found at (^8^). We included our training dataset in the Supplementary Material as the database file Table 1.xlsx. It contains the gene expression values data (columns A-O) together with the therapy result (Q column, 0 for nonresponsive and 1 for responsive case). Moreover, the database file includes information on the drug used (S column, PS341 for bortezomib and DEX for dexamethasone) and on the corresponding name of the GSM file with patient identification (U column). We considered information on 239 clinical tests. There were 169 tests with bortezomib, of which 84 were nonresponsive and 85 responsive, as well as 70 tests with dexamethasone, of which 42 were nonresponsive and 28 responsive.

Our approach is based on processing gene expression values with a network of chemical oscillators optimized for correlations between a single gene expression value and the result of therapy. The information processing network takes gene expression values as the inputs. The network functionality is optimized for maximum correlations between the result of therapy and the number of oscillations observed on the output oscillator, which is regarded as the network answer. The ideal network should simultaneously process information coming from many genes. However, it has been observed that in the case of many inputs, the evolutionary optimization of an information processing chemical network is long and ineffective because there are many local optima. In order to simplify the numerical simulations, we restricted our attention to networks made of three oscillators that process the expression data of a single gene. A typical optimized network returns ∼ 68% accuracy of prediction if for a given gene expression level, the therapy using bortezomib or dexamethasone is an effective treatment for multiple myeloma or not. We combined answers of single gene networks and made a concilium of networks based on the majority voting strategy. If such a strategy is applied to 15 considered genes, then we can predict the patient response to the drug therapy with an accuracy of ∼85% measured on the training dataset.

The paper is organized as follows: in Section 2 we define the procedure of input data normalization, introduce a 3-oscillator network and describe its optimization method. In the next section, we present optimization results and give the parameters of the best networks that correlate the individual gene expression values with the drug effectiveness. These networks are used for the concillum deciding if the drug therapy can be effective.

## 2 The Clinical Data and Their Classification

In the available dataset *R* = {*r*_*k*_, *k* = 1, 239} we have 239 patient records and each record *r*_*k*_ contains information on the expression values of *i* = 15 genes (*e*_*i*,*k*_, *i* = 1, 15) listed in Table 1. Moreover, Table 1 gives the maximum (*Max*_*k*_(*e*_*i*,*k*_)) and the minimum (*Min*_*k*_(*e*_*i*,*k*_)) gene expression values for each gene in *R*. The outcome of the therapy *z*_*k*_ ∈ {0, 1}, where 0 stands for nonresponsive therapy and 1 denotes responsive one, can be regarded as the record type. Therefore, a record *r*_*k*_ has the form of 16-tuple: *r*_*k*_ = (*e*_1,*k*_, …, *e*_15,*k*_, *z*_*k*_). The problem of deciding if the therapy can be effective for a given patient reduces to the problem of finding an algorithm that gives the correct record type *z* provided that the predictor values {*e*_*i*_, *i* = 1, 15} are known. It can be noticed that on this test group of patients, two trivial algorithms: 1) always use drugs and 2) do not use drugs because they will not help lead the correct therapy results in 47 and 53% cases respectively.

**FIGURE 1:**
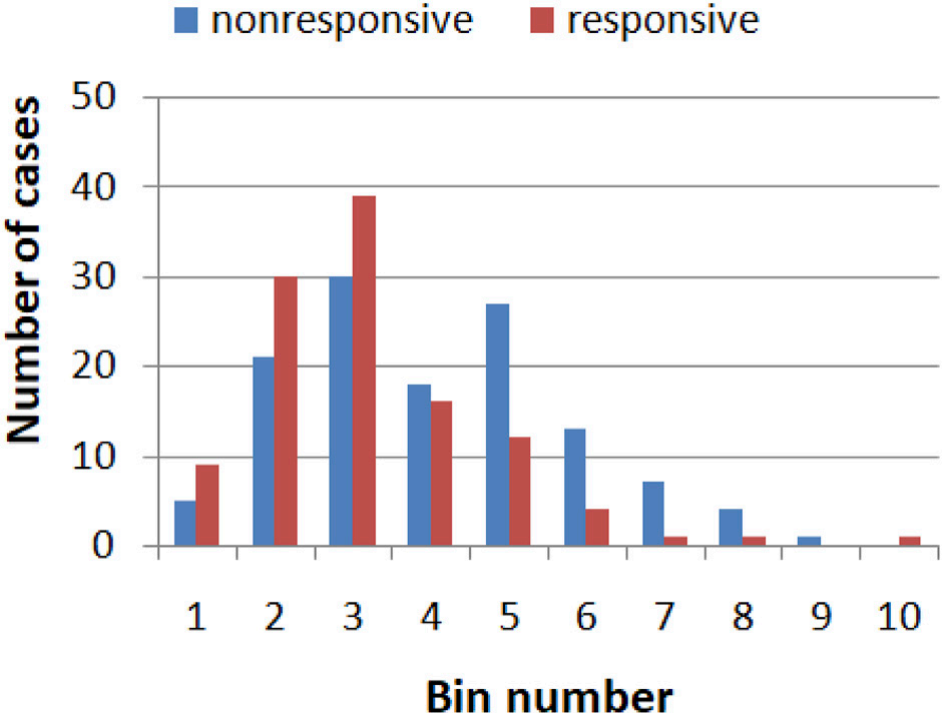
The histogram showing correlation between the expression of the RPS7 gene and responsive (red)/nonresponsive (blue) results of drug therapy. The range of gene expression values corresponding to each bin are given in Table 2.

The values of a single gene expression weakly correlate with the efficiency of the drug therapy. Figure 1 illustrates the correlations between the expression of the RPS7 gene and the responsive/nonresponsive results observed in clinical tests. The whole range of gene expression values is divided into 10 subintervals (bins) of the same length, and their ranges are given in Table 2. It can be noticed that if the gene expression value of RPS7 is within bins no. 1,2,3 or 10, then the probability of successful therapy is higher than its failure. On the other hand, if the expression of the RPS7 gene is above 4,445 but below 11,065 then it is more likely that multiple myeloma will not respond to the drug. Using this rule, we can plan the therapy with an accuracy of 62.3%, which is much higher than that of the trivial algorithms mentioned above. Of 239 cases included in the dataset R, we obtained 70 correctly determined nonresponsive cases and 79 correctly determined responsive ones. We also observed 56 wrongly determined nonresponsive cases and 34 wrongly determined responsive ones. In the following, we show how this accuracy can be improved with simple classification networks based on interacting chemical oscillators.

**TABLE 2:** The range of gene expression values in the bins considered in the histogram shown in Figure 1.

Bin number	Range of gene expression values in the Bin
1	1,142.4–2,244.9
2	2,244.9–3,347.4
3	3,347.4–4,449.9
4	4,449.9–5,552.4
5	5,552.4–6,654.8
6	6,654.8–7,757.3
7	7,757.3–8,859.8
8	8,859.8–9,962.3
9	9,962.3–11,064.8
10	11,064.8–12,167.3

It can be seen in Table 1 that the expression values significantly differ between genes. In order to unify the data for each gene we normalized the set of gene expression values *e*_*i*,*k*_ for all 239 patients using the formula:

$$p_{i,k}=\frac{e_{i,k}-Min_{k}\left(e_{i,k}\right)}{Max_{k}\left(e_{i,k}\right)-Min_{k}\left(e_{i,k}\right)}$$ (1)

**FIGURE 2:**
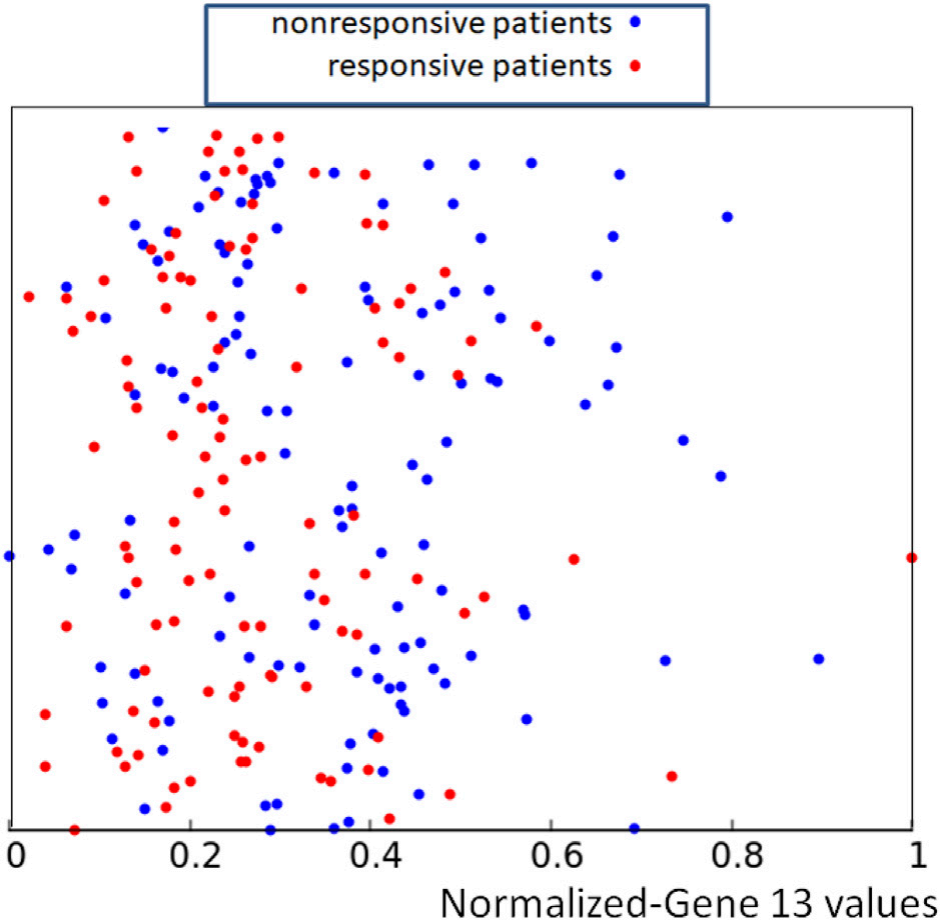
The distribution of normalized values of the RPS7 gene expression corresponding to responsive (red) and nonresponsive (blue) results of drug therapy respectively. The values of *p*_13,*k*_ are represented by the x-coordinate of marked points. The y-coordinate was randomly generated to differentiate points.

**FIGURE 3:**
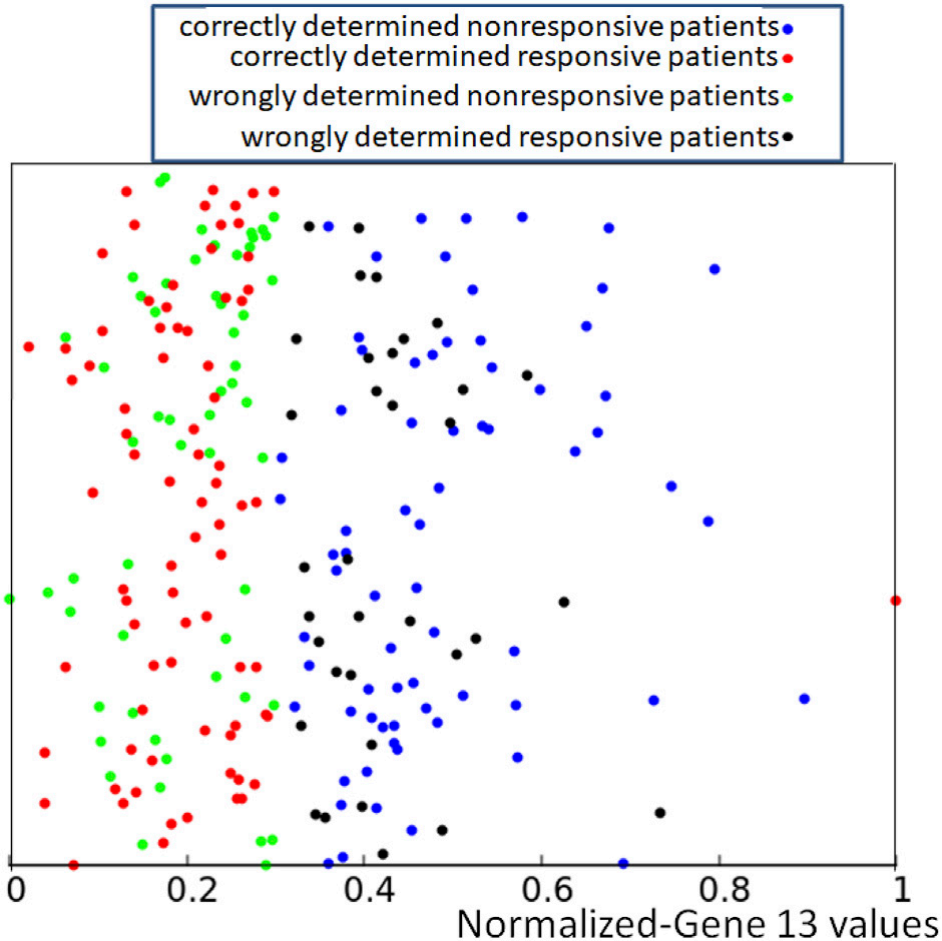
The distribution of normalized values of correctly and wrongly determined therapies using the majority rule based on the histogram of Figure 1. Red and blue points mark correctly determined responsive and nonresponsive records respectively. Black and green points mark wrongly determined responsive and nonresponsive records. The values of *p*_13,*k*_ are represented by the x-coordinate of marked points. The y-coordinate was randomly generated to differentiate points and it is the same as in Figure 2.

As an example the data {*p*_13,*k*_, *k* = 1, 239} are illustrated in Figure 2. For clearer presentation of data we show points {(*p*_13,*k*_, *y*_*k*_), *k* = 1, 239} where *y*_*k*_ is a random number in the interval [0, 1]. The random y-coordinates *Y* = {*y*_*k*_, *k* = 1, 239} were used to make the distribution of the training data easier to visualize, but of course, only the x-coordinate has a medical meaning. The same set of random y-coordinates *Y* was used to define points illustrated in Figures 3, 5, 7. The red and blue colors differentiate responsive and nonresponsive results of drug therapy, respectively. It can be seen that points corresponding to responsive and nonresponsive cases are not separated by the x-coordinate, related to the expression of gene no.13.

If we apply the rule following from the histogram shown in Figure 1 to the dataset of normalized data *Q* = {*q*_*k*_, *k* = 1, 239} where *q*_*k*_ = (*p*_1,*k*_, …, *p*_15,*k*_, *z*_*k*_) we obtain the distribution of correctly and wrongly determined results of drug therapy illustrated in Figure 3.

In the following, we consider the classification of the dataset *Q* using a network of interacting chemical oscillators. The idea of such computing was presented in the number of our papers (Gizynski and Gorecki, 2017a; Gizynski et al., 2017; Gorecki and Bose, 2020). We assume that there is a factor that controls oscillators and can inhibit oscillatory behavior. Such assumption is supported by the properties of Belousov Zabhotinsky(BZ) reaction (Belousov, 1959; Zhabotinsky, 1964) that has been widely used as a medium for chemical computing (Tóth and Showalter, 1995; Adamatzky et al., 2005; Yoshikawa et al., 2009; Gorecki et al., 2015; Dueñas-Díez and Pérez-Mercader, 2019; Proskurkin et al., 2020). The BZ-reaction is an oscillatory catalytic process (Epstein and Pojman, 1994). Among its reagents, we can distinguish *HBrO*_2_ acting as the reaction activator and *Br* ^−^ ions that are reaction inhibitors. It has been observed that for specific catalysts (for example, the ruthenium complex *Ru*(*bpy*)_3_), the reaction is photosensitive (Kuhnert, 1986; Kuhnert et al., 1989). The illumination with a blue light generates *Br* ^−^ ions that suppress oscillations (Kádár et al., 1997). The Oregonator model with two variables *u* and *v* representing concentrations of *HBrO*_2_ and the oxidized form of the catalyst respectively is described by the equations (Rovinskii et al., 1984; Adamatzky et al., 2005):

$$\frac{du}{dt}=\frac{1}{\varepsilon }\left(u-u^{2}-\left(fv+\phi \left(t\right)\right)\frac{u-q}{u+q}\right)$$ (2)

$$\frac{dv}{dt}=u-v$$ (3)

The time evolution of a medium where BZ-reaction proceeds are determined by the values of parameters: *f*, *q*, and *ɛ*. The parameter *ɛ* sets up the ratio of time scale between variables *u* and *v*, *q* is the scaling constant, and *f* is the stoichiometric coefficient. The time-dependent function *ϕ*(*t*) is related to the medium illumination. The values of parameters *f*, *q* and *ɛ* can be selected such that for small *ϕ*(*t*) there are oscillations in *u* and *v*, but for a large *ϕ* the system converges to a stable stationary state (Kádár et al., 1997; Gorecki et al., 2014). Therefore, the value of *ϕ* can be interpreted as the light intensity in the Ru-catalyzed BZ-reaction. It can be used as an external factor to suppress oscillations or restore them. In the following, we used a modified Oregonator model to describe the time evolution of the considered computing oscillator networks.

There are a few arguments for the selection of the 2-variable Oregonator model. First, it provides a more realistic description of an oscillator network based on BZ-reaction than the oversimplified event-based-model used in early studies on computing networks of oscillators (Gizynski and Gorecki, 2017a; Gizynski et al., 2017). For example, the Oregonator model takes into account the effect of combined excitation of an oscillator by a few neighbors, which is missing in the event-based-model. Second, the model is still computationally simple, and it allows to perform a complex evolutionary optimization involving a huge number of evaluations of network evolution. Moreover, despite its simplicity, it provides a better than qualitative description of many phenomena related to BZ-reaction. It correctly describes the oscillation period as a function of reagent concentration and also can be used to model non-trivial phenomena like the migration of a spiral in an electric field (Sutthiopad et al., 2014) or reaction of a propagating pulse to time-dependent illumination (Tanaka et al., 2007). Of course, a model with a larger number of variables gives a more realistic description of BZ-reaction but, on the other hand, requires a more precise model of interactions between oscillators.

**FIGURE 4:**
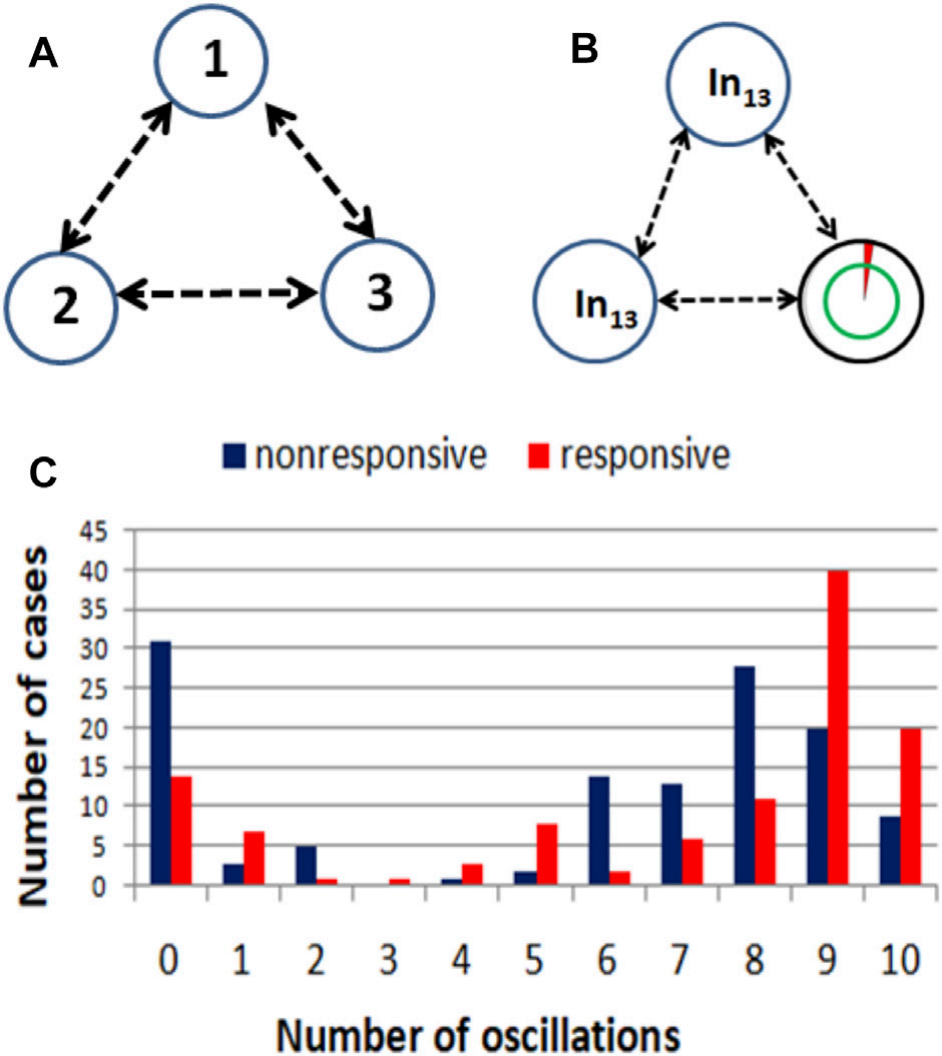
**(A)** An example illustrating the idea of a computing oscillator network. **(B)** The structure a network that produces a high correlation between the RPS7 gene expression and the result of drug therapy. The symbol *In*_13_ marks input oscillators. The rightmost oscillator is a normal one, and it is also the output oscillator what is indicated with a double circle. **(C)** Correlations between the success of therapy and the number of activator maxima observed on the output oscillator.

An example illustrating the idea of a considered computing oscillator network is illustrated in Figure 4A (Gorecki and Bose, 2020). The network is formed by three coupled oscillators marked by circles. We assume that the output information can be extracted from the observation of the network evolution during the time interval [0, *t*_max_]. More precisely, the output information is coded in the number of activator maxima that are higher than a threshold value (in our study, this is 0.05) that are observed within the time interval [0, *t*_max_] on a selected oscillator of the network. We consider time-dependent illumination *ϕ*_*j*_(*t*) of the oscillator *#j* in the form:

$$\phi_{j}\left(t\right)=0.1\cdot \left(\right.1.001+\tanh\left(-10\left(t-t_{\mathrm{illum}}\left(j\right)\right)\right)$$ (4)

This functional dependence is the same for every oscillator; however, the value of parameter *t*_*illum*_(*j*) differs between oscillators. At the beginning of evolution, the inhibiting factor is high, which means that the oscillator is in a stationary state. For times *t* > *t*_*illum*_(*j*) oscillations on the *j*th oscillator appear. At long times the value of *ϕ*_*i*_(*j*) approaches 0.0001. For such illumination, Eqs 2, 3 produce oscillations characterized by the period of 8.2 time unit.

The use of illumination time (or, in general, the inhibition time for an oscillator) *t*_*illum*_ to influence oscillators is inspired by our experiments in which oscillations in individual BZ-droplets were controlled by blue LEDs (Gizynski and Gorecki, 2017b). In these experiments, we used just two illumination intensities: a low one for which the droplet was oscillating and a high one inhibiting oscillations. The transitions between the steady state and oscillations predicted by the 2-variable Oregonator model were in qualitative agreement with observations. For further studies, it will be interesting to consider more complex forms of time-dependent illumination because it has been observed that the time evolution of BZ-medium depends on the rate of changes in applied illumination (Tanaka et al., 2007).

Oscillators that form a computing network are of one of two types: the input oscillators and the normal ones (Gizynski and Gorecki, 2017a; Gizynski et al., 2017; Gorecki and Bose, 2020). If the *j*th oscillator is considered as a normal one, then the value of *t*_*illum*_(*j*) is fixed. If this oscillator is consider as the input of a predictor *p*_*i*_ then the value of *t*_*illum*_(*j*) is functionally related to *p*_*i*_. For our analysis, we assume that the function has the form:

$$t_{\mathrm{illum}}\left(j\right)=t_{\mathrm{start}}+\left(t_{\mathrm{end}}-t_{\mathrm{start}}\right)*p_{i}$$ (5)

The transformation given by Eq. 1 with parameters listed in Table 1 normalized the data included in the test dataset *R* to the interval [0, 1]. But we would like to know if the therapy is effective for any potential patient. Assume that the measured gene expression values are ${\tilde{e}}_{i}$ , *i* = 1, 15. It can happen that some of ${\tilde{e}}_{i}$ are outside of the range listed in Table 1. Applying transformation (Mulligan et al., 2007) we obtain the corresponding ${\tilde{p}}_{i}$ outside [0, 1]. Nevertheless, according to Eq. 5 such input values also produce meaningful values of *t*_*illum*_(*j*) that are accepted by gene information processing oscillator networks discussed below.

There is the output oscillator in the network. For fixed parameters describing the network, we select the output oscillator as the one that produces the highest accuracy of predicting record types for the dataset used for network training. In order to determine the network accuracy, we applied the following method. The network time evolution is simulated for all records of *Q*. For each oscillator and for each number of activator maxima, we formulate the relationship between the number of activator maxima and the outcome of therapy based on the majority of cases. The oscillator for which the number of errors is minimized is regarded as the output one.

The coupling between oscillators, indicated by two direction arrows in Figure 4A is achieved by reactions that extend the original Oregonator model. We assume that the coupling is of the activatory type and occurs via the exchange of reactor activators between oscillators (Gorecki and Bose, 2020; Bose and Gorecki, 2022). Let *U*_*i*_ denotes the activator of the *ith* oscillator. The exchange is described by reactions:

$$U_{j}+B_{j}\to U_{i}+C_{i}$$ (6)

$$U_{i}+B_{i}\to U_{j}+C_{j}$$ (7)

thus the coupling between oscillators is symmetric. We also assumed that the activator of each reaction can spontaneously decay in the process:

$$U_{j}+D_{j}\to products$$ (8)

The symbols *B*, *C* and *D* appearing in the reactions above denote other molecules involved in these reactions. The concentrations of *B* and *D* (*b*, *d*) were assumed to be high with respect to the concentrations of activator and inhibitor, and hence their concentration was treated to be constant. If *k*_*B*_ and *k*_*A*_ are the reaction rate constants of reactions corresponding to coupling and decay respectively then the decrease in activator concentrations is described by the terms: *k*_*B*_ *b*_*j*_ *u*_*j*_, *k*_*B*_ *b*_*i*_ *u*_*i*_, and *k*_*A*_ *d*_*j*_ *u*_*j*_ respectively. Having in mind high concentrations of *B*_*j*_ and *D*_*j*_ we can write those as *βu*_*j*_, *βu*_*j*_ and *αu*_*j*_ where *α* and *β* are parameters with values controlled by *b* and *d*.

On the basis of the above assumptions we can formulate the following equations describing the time evolution of the network:

$$\frac{du_{j}}{dt}=\frac{1}{\varepsilon }\left(u_{j}-u_{j}^{2}-\left(fv_{j}+\phi_{j}\left(t\right)\right)\frac{u_{j}-q}{u_{j}+q}\right)-\left(\alpha +\beta \underset{i=1,m}{\sum }s_{j,i}\right)u_{j}+\beta \underset{i=1,m}{\sum }s_{j,i}u_{i}$$ (9)

$$\frac{dv_{j}}{dt}=u_{j}-v_{j}$$ (10)

where i,j represent the *jth* and *ith* oscillator and *m* is the number of oscillators in the network. The variables *u*_*j*_ and *v*_*j*_ denote the concentration of an activator *U*_*j*_ and an inhibitor *V*_*j*_ respectively. The symbols *s*_*j*,*i*_ are defined as:

*s*_*j*,*i*_ = 0 if *j* = *i* or if *j* ≠ *i* and oscillators *#j* and *#i* do not interact,

*s*_*j*,*i*_ = 1 if *j* ≠ *i* and oscillators *#j* and *#i* do interact.

In our simulations we used the following set of Oregonator model parameters: *ɛ* = 0.3, *q* = 0.002, *f* = 1.1. For *ϕ*_*j*_(*t* = 0) = 0.2 and these parameter values the stable steady state of Eqs 2, 3 is *u*_*j*_ = 0.00204 and *v*_*j*_ = 0.00204.

In order to define an information processing chemical oscillator network we have to specify many parameters: the number of oscillators *m*, the geometry of their connections (*s*_*j*,*i*_), location of input and normal oscillators, all parameters for a model of chemical oscillations (*ɛ*, *q* and *f*), rates for reactions responsible for interactions between oscillators (*α*, *β*) the observation time *t*_max_, illumination times for all normal oscillators *t*_*illum*_(*i*) and parameters *t*_*start*_ and *t*_*end*_ that translate an input value into the illumination of an input oscillator (cf. Eq. 5). The problem is even more complicated as we can consider other models of chemical oscillators than Oregonator, different functions linking input values with the time interval within which the inhibiting factor is applied, and various models for coupling between oscillators. We do not know any algorithm that allows for a straightforward design of the optimum oscillator network for a given problem. Still, we can apply a parameter optimization algorithm to a training dataset in the hope it produces a network that gives a reasonable solution to the problem. However, optimization of all parameters mentioned above represents a computational problem of very high complexity. Before starting the optimization, we introduced a number of simplifications:

(1) we restricted our attention to classifiers formed by *m* = 3 oscillators,
(2) we assumed that each oscillator interacted with all others, so *s*_*j*,*i*_ ≡ 1 for *j* ≠ *i*. The geometry of such network is illustrated in Figure 4A.
(3) There has to be an input oscillator in the network and a normal one. Without the input oscillator, the network returns the same answer on all inputs. Without the normal oscillator, the network evolves like a single oscillator. Keeping in mind the symmetry of the considered network, we can assume that the oscillator #1 is the input oscillator and the oscillator #3 is a normal one. The role of the oscillator #2 is the subject of optimization.

After these simplifications the network is fully characterized by *t*_max_, *α*, *β*, *t*_*start*_, *t*_*end*_, *t*_*illum*_ (3), the role of oscillator #2 and if it is the normal one, its illumination time *t*_*illum*_ (2). We optimized the values of these parameters using *Q* as the training dataset. The fitness function of evolutionary optimization was the maximum accuracy between the network output coded in the number of activator maxima observed on one of the oscillators and the record type *z* (Gizynski and Gorecki, 2017a; Gizynski et al., 2017; Gorecki and Bose, 2020). The time evolution of networks was obtained by solving Eqs 9, 10 numerically using 5-th order Cash-Karp algorithm (Cash and Karp, 1990) with *dt* = 10^–3^ time steps. For a given network, all three oscillators were considered as potential candidates for output one. The oscillator that produced the highest accuracy on the training dataset was regarded as the output one. The applied evolutionary algorithm is a standard one (Goldberg, 1989), and it has been described in our previous papers (Gizynski and Gorecki, 2017a; Gizynski et al., 2017; Gorecki and Bose, 2020). The optimization started with 100 networks with randomly generated parameters. The next generation of networks included 5 top fit networks of the previous generation and 95 networks formed by recombination of parameters of two networks selected from 40 best networks of the previous generation. Each network obtained by recombination was then allowed to mutate. Mutations included the values of all parameters and the type of oscillator #2. The optimized network was obtained after 500 evolutionary steps.

## 3 Results

The evolutionary optimization described in the previous Section was used to design networks with a high correlation between the number of activator maxima observed on one of the oscillators and the result of drug therapy. As an example, we show such a network for linking the RPS7 normalized gene expression value with the success of therapy. For this input, the evolutionary optimization produced the network illustrated in Figure 4B. It is composed of two input oscillators, marked with *In*_13_, that accept the value of *p*_13,*k*_. The rightmost oscillator, marked with the double circle, is the output one. The circle marking this oscillator is also a base for a pie chart representing the ratio *t*_*illum*_ (3)/*t*_max_ by the surface of the red slice. Figure 4C shows the distribution of a number of activator maxima observed on the output oscillator for responsive and nonresponsive multiple myeloma treatment. On this basis, we can define the rule that ensures the highest accuracy on the training dataset *Q*. For this network, the rule is:

- if 1,3,4,5,9 or 10 activator maxima are observed on the output oscillator, then the patient belongs to the responsive group.
- if another number of activator maxima is observed, then unsuccessful treatment with the drugs is expected.

**FIGURE 5:**
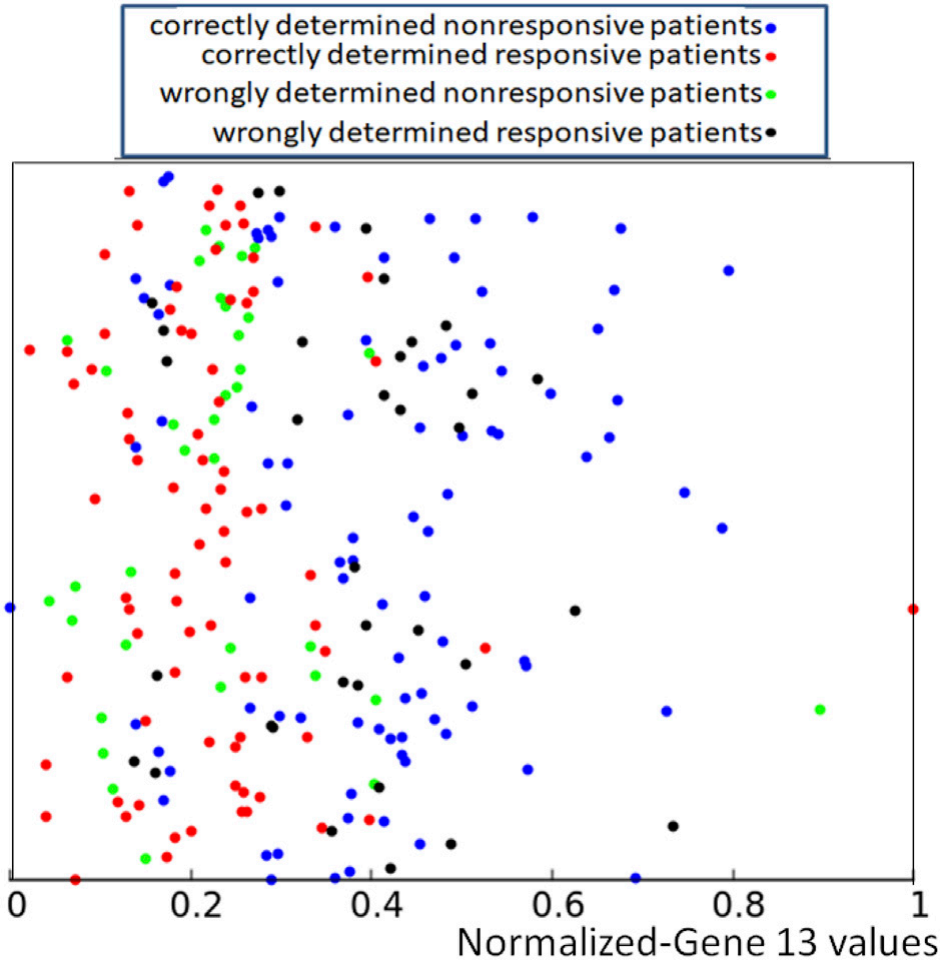
Location of correct and incorrect predictions on the result of drug therapy by a network of oscillators optimized for the RPS7 gene. The values of *p*_13,*k*_ are represented by the x-coordinate of marked points. The y-coordinate is randomly generated to differentiate points and is the same as in Figure 2.

If applied to the training dataset *Q*, this rule gives 71.1% of correct answers, which is 5% higher than the trivial rule based on the distribution of gene expression values (cf. Figure 1). Of 239 cases included in the dataset *Q*, we obtained 91 correctly determined nonresponsive cases and 79 correctly determined responsive ones. We also observed 35 wrongly determined nonresponsive cases and 34 wrongly determined responsive ones. The distribution of correctly and incorrectly classified points from the training dataset is illustrated in Figure 5. Here again, the y-coordinate is random, and it is the same as introduced to differentiate the values of *p*_13,*k*_ in Figure 2.

**FIGURE 6:**
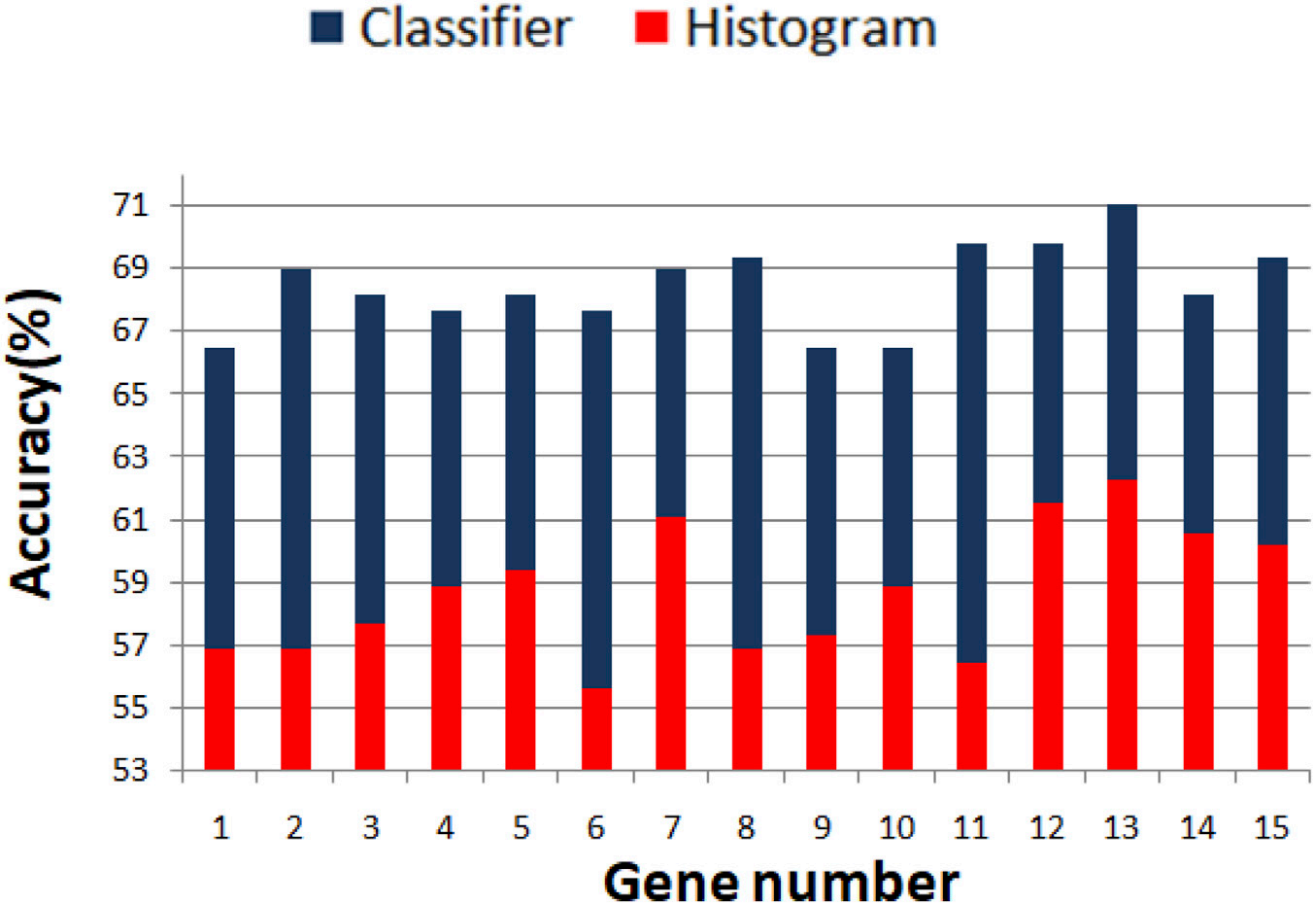
Comparison between the accuracy of predictions of the result of drug therapy based on the histogram of gene expression values (red bars) and the optimized network of oscillators (blue bars). The gene numbers correspond to these in Table 1.

We optimized 3-oscillator networks for all genes listed in Table 1. The parameters of all optimized networks are listed in Table 3. The gene numbers in Table 3, Table 4, and Figure 6 correspond to those in Table 1. The rules that translate the number of activator maxima on the output oscillator and the therapy result are given in Table 4. The accuracy of optimized information processing networks is shown in Figure 6, and in Table 4. It is in the range between 66.5% (genes SERP1, CXCL5 and IL15) to 71.1% (gene RPS7).

**TABLE 3:** The parameters of networks that give the best correlations between the number of activator maxima on the output oscillator and the therapy result.

Gene no.	*t*_max_	*t*_*start*_	*t*_*end*_	*α*	*β*	Input oscillators	Output oscillator	Normal oscillators	$t_{\mathrm{illum}}$ (i)
1	77.35	28.11	2.31	0.69	0.08	1	2	2, 3	*t*_*illum*_ (2) = 17.63
									*t*_*illum*_ (3) = 7.24
2	77.55	2.03	33.77	0.7	0.07	1	1	2, 3	*t*_*illum*_ (2) = 8.73
									*t*_*illum*_ (3) = 4.33
3	80	63.81	2.26	0.7	0.08	1, 2	1	3	*t*_*illum*_ (3) = 8.58
4	77.45	6.02	35.37	0.68	0.074	1	1	2,3	*t*_*illum*_ (2) = 8.26
									*t*_*illum*_ (3) = 2.84
5	80	2.45	68.46	0.62	0.06	1, 2	3	3	*t*_*illum*_ (3) = 4.34
6	80	2.1	42.37	0.7	0.09	1	1	2, 3	*t*_*illum*_ (2) = 2.58
									*t*_*illum*_ (3) = 12.11
7	80	1.87	69.8	0.63	0.06	1	2	2, 3	*t*_*illum*_ (2) = 3.51
									*t*_*illum*_ (3) = 5.28
8	70.3	1.82	32.68	0.70	0.081	1	1	2, 3	*t*_*illum*_ (2) = 2.64
									*t*_*illum*_ (3) = 10.68
9	74.63	2.49	28.12	0.70	0.08	1, 2	3	3	*t*_*illum*_ (3) = 10.56
10	80	2.56	29.14	0.7	0.06	1, 2	3	3	*t*_*illum*_ (3) = 4.33
11	80	2.74	29.05	0.69	0.07	1	2	2, 3	*t*_*illum*_ (2) = 3.16
									*t*_*illum*_ (3) = 7.25
12	70.79	70.65	1.57	0.59	0.09	1, 2	1	3	*t*_*illum*_ (3) = 13.42
13	80	2.48	51.09	0.7	0.07	1, 2	3	3	*t*_*illum*_ (3) = 2.0
14	80	36.28	2.79	0.7	0.08	1, 2	3	3	*t*_*illum*_ (3) = 4.39
15	75.71	62.57	3.52	0.66	0.08	1, 2	3	3	*t*_*illum*_ (3) = 0.78

**TABLE 4:** The rules that translate the number of activator maxima on the output oscillator and the effective therapy using bortezomib or dexamethasone drugs.

Input gene no.	Number of activator maxima for responsive therapy	Number of activator maxima for nonresponsive therapy	Accuracy %
1	0, 1, 3, 5, 7, 10	2, 4, 6, 8, 9, 11	66.5
2	3, 6, 7, 9, 10	1, 2, 4, 5, 8, 11	60.0
3	2, 6, 7, 8, 9, 11	1, 3, 4, 5, 10	68.2
4	1, 2, 3, 4, 7, 9	0, 5, 6, 8	67.7
5	1, 5, 7, 9	3, 4, 6, 8, 10, 11	68.2
6	2, 5, 6	0, 1, 3, 4, 7, 8, 9	67.7
7	2, 6,9	1, 3, 4, 5, 7, 8, 10, 11	69.0
8	0, 2, 4, 6, 7, 8, 9	1, 3, 5, 10	69.4
9	0, 2, 3, 5, 7, 8	1, 4, 6, 9, 10	66.5
10	1, 3, 6, 7, 10	2, 4, 5, 8, 9, 11	66.5
11	1, 2, 6, 7, 11	3, 4, 5, 8, 9, 10	69.8
12	2, 3	1, 3, 4, 5, 6, 7, 8	69.8
13	1, 3, 4, 5, 9, 10	0, 2, 6, 7, 8	71.1
14	0, 1, 2, 4, 7	3, 5, 6, 8, 9, 10, 11	68.2
15	3, 4	1, 2, 5, 6, 7, 8, 10	69.4

**FIGURE 7:**
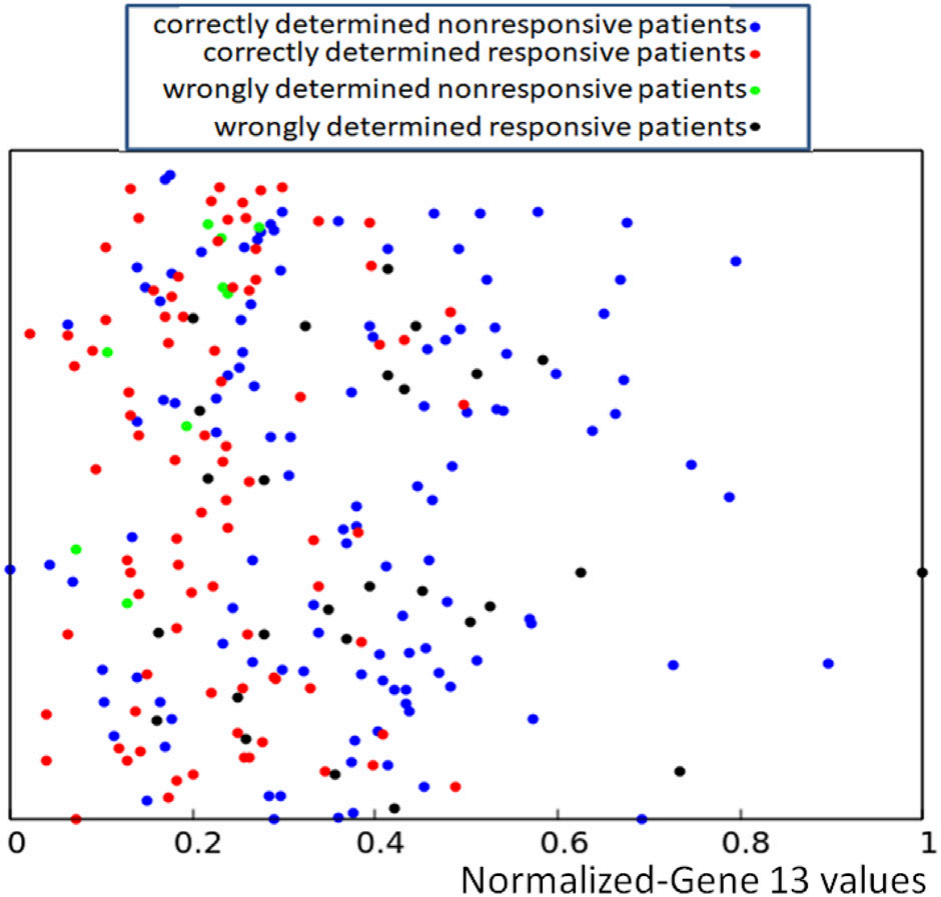
Location of correct and incorrect predictions on the result of drug therapy predicted by a concilium of networks of oscillators optimized for all genes. The values of *p*_13,*k*_ are represented by the x-coordinate of marked points. The y-coordinate is the same as in Figure 2.

To increase the accuracy in determining the success or failure of drug therapy, we called a concilium of optimized networks. Each member of concilium is a network specialized in finding correlations between the expression value of one gene and the success of drug therapy and has one vote. The final decision is taken on the basis of majority voting. The accuracy of determining if the therapy using the bortezomib or dexamethasone drugs is efficient or not for different majority rules applied to the concilium is shown in the second column of Table 5. We can see that the decision based on the opinions of more than half of concilium members is accurate for almost 85% of cases included in the training dataset. Of 239 cases included in the dataset *Q* we obtained 117 correctly determined nonresponsive cases and 86 correctly determined responsive ones. We also observe 9 wrongly determined nonresponsive cases and 27 wrongly determined responsive ones. Their distribution is illustrated in Figure 7.

**TABLE 5:** The accuracy of determination if the therapy using the bortezomib or dexamethasone drugs is efficient for different majority rules. The results for the optimized network and the network with modified parameters (cf. Table 6) are compared.

The majority rule	The concillum accuracy (%)	The accuracy of concillium for bortezomib cases only (%)	The accuracy of concillium for dexamethasone cases only (%)	The accuracy of concillium with modified networks (%)
14 or more	7.1	7.6	5.7	8.3
votes for				
13 or more	17.1	19.5	11.4	17.1
votes for				
12 or more	30.9	34.3	22.8	28.0
votes for				
11 or more	43.0	43.7	41.4	40.5
votes for				
10 or more	61.5	62.1	60	56.9
votes for				
9 or more	77.4	78.6	74.2	69.0
votes for				
8 or more	84.9	85.7	82.8	82.8
votes for				

But do we need oscillator networks? Correlations between the values of single gene expression corresponding to responsive and nonresponsive cases from the training dataset and the results of drug therapy can be extracted from the histograms of single gene expression values, as it was done for the RPS7 gene (cf. Figure 1). For this gene, the accuracy of such a method is 62.3% which is not much lower than that of the optimized classifier (71.1%). So why not try to make the conciluim based on histograms for all genes? We have tested such an approach by dividing the whole range of gene expression values into 10 subintervals and introducing the rule based on the majority of cases in subintervals for each gene. Next, we applied the majority voting strategy for all records of *Q*. The accuracy of such concilium was 66.9%. Of 239 cases included in the dataset *Q*, we obtained 100 correctly determined nonresponsive cases and 60 correctly determined responsive ones. We also observed 26 wrongly determined nonresponsive cases and 53 wrongly determined responsive ones. Therefore the accuracy of such concilium is much smaller than the concilium based on networks of oscillators optimized for correlations between the single gene expression value and the outcome of therapy.

## 4 Conclusion and Discussion

In this paper we discussed the application of information processing network formed by chemical oscillators for determination of the outcome of the multiple myeloma therapy with bortezomib or dexamethasone drugs. The network input information comes form the gene expression values. Information was processed by simple networks, each made of 3 oscillators. Each network was optimized to find correlations between the expression value of a particular gene and the outcome of the therapy. Individual classifiers gave the accuracy in the range between 66.5 and 71.1% (cf. Figure 7). To improve the determination of the therapy outcome we considered the concilium of 15 classifiers and accepted the majority decision. Such strategy increased the accuracy to almost 85%, which seems to be a promising result for the further development of the method.

In the mid columns of Table 5 we presented the accuracy of the concilium method based on classifiers optimized for the whole training dataset to therapies in which one of the drugs (bortezomib or dexamethasone) was used. The idea was to find if the therapy prediction accuracy depends on the drug. The results are similar, so we can conclude that for both drugs, the patient genetic profile is similarly correlated with the therapy success. It would be interesting to make a similar concilium separately for each drug, but the solution to this problem requires a much larger database of clinical trials than the one we had access to.

To check how sensitive to fluctuations of parameters are the results produced by the concilium formed of optimized networks, we considered random modifications in parameter values. For each optimized network, we selected one parameter at random and decreased or increased its value by ± 1%. The details on applied modifications and their influence on the accuracy of each network are given in Table 6. In all cases, the accuracy decreased by 2–3%. A similar decrease of accuracy is observed for the decision of concilium (82.8% for the concilium of modified networks). Still, such accuracy is high enough to claim that concilium strategy for determination of drug effectiveness is robust to random changes in parameters and fluctuations in the medium.

**TABLE 6:** The accuracy of modified oscillator networks for correlations between the gene expression value and the therapy result. The table also defines modification introduced to the optimized network.

Gene no.	New parameter = old parameter ±1% of old parameter	Output oscillator	Accuracy (%)
1	*t*_max_ + 1%	2	64.8
2	*β* + 1%	1	66.9
3	*β* − 1%	1	66.9
4	*t*_*start*_ + 1%	1	62.7
5	$t_{\mathrm{illum}}^{3}-1\%$	2	65.6
6	*t*_*end*_ + 1%	1	64.0
7	*β* + 1%	2	67.3
8	$t_{\mathrm{illum}}^{2}-1\%$	1	66.9
9	*α* − 1%	2	62.3
10	$t_{\mathrm{illum}}^{3}+1\%$	3	63.1
11	$t_{\mathrm{illum}}^{2}+1\%$	2	62.3
12	*t*_*end*_ + 1%	3	69.4
13	*β* − 1%	3	69.8
14	*t*_max_ + 1%	2	66.1
15	$t_{\mathrm{illum}}^{3}-1\%$	3	69.0

We can suggest two ways in which the accuracy in the determination of therapy effectiveness can be increased:

One of them is to consider the voting strategy with more complex networks formed by a larger number of oscillators that are used to determine correlations between a single gene expression value and the therapy outcome. One can expect that “wiser” members of concilium can produce more accurate answers. However, the strategy of employing top specialists does not guarantee top results. Our simulations have shown that the synergy between concilium members is also important and should be taken into account. We continued optimization for some networks processing gene expression values and obtained higher accuracy than that listed in Table 4. However, if we replaced the optimized network for the gene SERP1 that led to the accuracy of 66.5% (cf. #1 in Table 3) by a wiser member (accuracy 67.7%, *t*_max_ = 80, *t*_*start*_ = 3.52, *t*_*end*_ = 32.23, = 0.71, *β* = 0.09, normal oscillators 2 and 3, $t_{\mathrm{illum}}^{2}=3.38$ , $t_{\mathrm{illum}}^{3}=4.24$ ) than the accuracy of concilium decreased to 83.6%.

**FIGURE 8:**
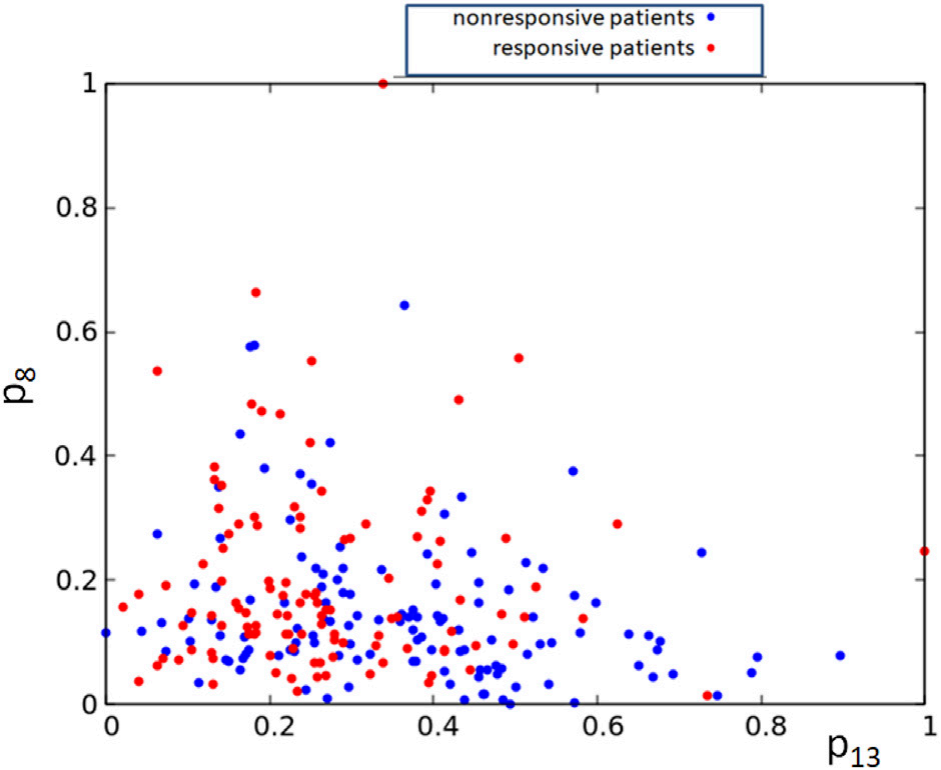
The distribution of normalized expression values of the RPS7 and CFLAR genes corresponding to responsive (red) and nonresponsive (blue) results of drug therapy respectively. The point coordinates are (*p*_13,*k*_, *p*_8,*k*_), *k* = 1, 239.

Alternatively, one can consider networks that are processing expression values for more than a single gene. However, the pairs of gene expression values corresponding to responsive and nonresponsive therapies do not show clear separation in the square [0, 1] × [0, 1] (cf. Figure 8). Therefore, it can be anticipated that a large oscillator network is necessary for the data classification, and its optimization will be numerically complex.

Finally, let us make more general comments on the importance of the presented results:

First, they demonstrate a high computing potential of networks composed of interacting oscillators. The distributions of gene-expression values corresponding to responsive and non-responsive patients do not show a clear separation, as illustrated in Figure 2 and indicated by the histogram in Figure 1. We can expect that classification of these data with classical neural networks is inefficient and requires a large number of nodes. We demonstrated that the cases separation with a reasonable accuracy exceeding 65% can be done with a network of just 3 oscillators. Suppose the effectiveness of oscillator networks is confirmed on other problems. Then, as a natural development of this approach, we can expect a new class of integrated circuits made with semiconductors with easy control of the geometry of interactions and parameters of oscillators. They can operate similar to Intel Neural Compute Stick designed to support computation with classical neural networks^9^.

Second, the semiconductor devices work properly in a narrow range of temperatures around the room ones, whereas the range of conditions in which chemical oscillations are observed is much wider. Therefore, we can think of designing chemical computers for the specific environment they are supposed to function, for example, for space research applications. Furthermore, chemical computers operate on the energy of their reagents, so they do not need additional energy supply.

## Data Availability

The original contributions presented in the study are included in the article/Supplementary Material, further inquiries can be directed to the corresponding author.

## Footnotes

1
https://www.cancertherapyadvisor.com/home/cancer-topics/hematologic-cancers/hematologic-cancers-treatment-regimens/multiple-myeloma-treatment-regimens/

2
https://ashpublications.org/blood/article/109/8/3177/23711/Gene-expression-profiling-and-correlation-with

3
https://www.ncbi.nlm.nih.gov/geo/query/acc.cgi?acc=GSE9782

4
https://www.ncbi.nlm.nih.gov/gene

5
https://www.qiagen.com/us/products/discovery-and-translational-research/dna-rna-purification/rna-purification/total-rna/rneasy-kits/

6
https://en.wikipedia.org/wiki/DNA_microarray

7
https://ashpublications.org/blood/article/109/8/3177/23711/Gene-expression-profiling-and-correlation-with

8
https://www.ncbi.nlm.nih.gov/geo/query/acc.cgi?acc=GSE9782

9
https://www.intel.com/content/www/us/en/developer/tools/neural-compute-stick/overview.html

## References

[B1] Adamatzky A. De Lacy Costello B. Asai T. (2005). Reaction–diffusion Computers. New York, NY, USA: Elsevier.

[B2] Adamatzky A. Holley J. Bull L. De Lacy Costello B. (2011). On Computing in Fine-Grained Compartmentalised Belousov-Zhabotinsky Medium. Chaos, Solit. Fractals 44, 779–790. 10.1016/j.chaos.2011.03.010

[B3] Adamatzky A. Holley J. Dittrich P. Gorecki J. De Lacy Costello B. Zauner K. P. (2012). On Architectures of Circuits Implemented in Simulated Belousov-Zhabotinsky Droplets. Biosystems 109, 72–77. 10.1016/j.biosystems.2011.12.007 22261640

[B4] Belousov B. P. (1959). Collection of Short Papers on Radiation Medicine. Moscow: Medgiz, 145–152.

[B5] Bose A. Gorecki J. (2022). Computing with Networks of Chemical Oscillators and its Application for Schizophrenia Diagnosis. Front. Chem. 10. 10.3389/FCHEM.2022.848685 PMC8966613 35372264

[B6] Cash J. R. Karp A. H. (1990). A Variable Order Runge-Kutta Method for Initial Value Problems with Rapidly Varying Right-Hand Sides. ACM Trans. Math. Softw. 16, 201–222. 10.1145/79505.79507

[B7] Dueñas-Díez M. Pérez-Mercader J. (2019). How Chemistry Computes: Language Recognition by Non-Biochemical Chemical Automata. From Finite Automata to Turing Machines. iScience 19, 514–526. 10.1016/j.isci.2019.08.007 31442667 PMC6710637

[B8] Epstein I. R. Pojman J. A. (1994). Introduction to Nonlinear Chemical Dynamics: Oscillations, Waves, Patterns, and Chaos. New York, NY, USA: Oxford University Press.

[B9] Feynman R. P. Hey T. Allen R. (2000). Feynman Lectures on Computation. Boulder, Colorado, USA: CRC Press.

[B10] Field-Smith A. Morgan G. J. Davies F. E. (2006). Bortezomib (Velcade?) in the treatment of multiple myeloma. Ther. Clin. Risk Manag. 2 (3), 271–279. 10.2147/tcrm.2006.2.3.271 18360602 PMC1936263

[B11] Gizynski K. Gorecki J. (2017). Cancer classification with a network of chemical oscillators. Phys. Chem. Chem. Phys. 19, 28808–28819. 10.1039/c7cp05655a 29051945

[B12] Gizynski K. Gorecki J. (2017). Chemical memory with states coded in light controlled oscillations of interacting Belousov-Zhabotinsky droplets. Phys. Chem. Chem. Phys. 19 (9), 6519–6531. 10.1039/c6cp07492h 28197558

[B13] Gizynski K. Gruenert G. Dittrich P. Gorecki J. (2017). Evolutionary Design of Classifiers Made of Droplets Containing a Nonlinear Chemical Medium. Evol. Comput. 25, 643–671. 10.1162/evco_a_00197 27728772

[B14] Goldberg D. E. (1989). Genetic Algorithms in Search, Optimization and Machine Learning. Boston, MA: Addison-Wesley Longman Publishing Co., Inc.

[B15] Gorecka J. Gorecki J. (2006). Multiargument logical operations performed with excitable chemical medium. J. Chem. Phys. 124, 084101. 10.1063/1.2170076 16512702

[B16] Gorecki J. Bose A. (2020). How Does a Simple Network of Chemical Oscillators See the Japanese Flag? Front. Chem. 8, 580703. 10.3389/fchem.2020.580703 33240845 PMC7680917

[B17] Gorecki J. Gizynski K. Guzowski J. Gorecka J. N. Garstecki P. Gruenert G. (2015). Chemical computing with reaction-diffusion processes. Phil. Trans. R. Soc. A 373, 20140219. 10.1098/rsta.2014.0219 26078345

[B18] Gorecki J. Gorecka J. N. Adamatzky A. (2014). Information coding with frequency of oscillations in Belousov-Zhabotinsky encapsulated disks. Phys. Rev. E 89, 042910. 10.1103/PhysRevE.89.042910 24827316

[B19] Hideshima T. Bergsagel P. L. Kuehl W. M. Anderson K. C. (2004). Advances in biology of multiple myeloma: clinical applications. Blood 104, 607–618. 10.1182/blood-2004-01-0037 15090448

[B20] Holley J. Adamatzky A. Bull L. De Lacy Costello B. Jahan I. (2011). Computational modalities of Belousov-Zhabotinsky encapsulated vesicles. Nano Commun. Netw. 2, 50–61. 10.1016/j.nancom.2011.02.002

[B21] Kádár S. Amemiya T. Showalter K. (1997). Reaction Mechanism for Light Sensitivity of the Ru(bpy)32+-Catalyzed Belousov−Zhabotinsky Reaction. J. Phys. Chem. A 101, 8200–8206. 10.1021/jp971937y

[B22] Kuhnert L. (1986). A new optical photochemical memory device in a light-sensitive chemical active medium. Nature 319, 393–394. 10.1038/319393a0

[B23] Kuhnert L. Agladze K. I. Krinsky V. I. (1989). Image processing using light-sensitive chemical waves. Nature 337, 244–247. 10.1038/337244a0

[B24] Lesko L. J. Woodcock J. (2004). Translation of pharmacogenomics and pharmacogenetics: a regulatory perspective. Nat. Rev. Drug Discov. 3, 763–769. 10.1038/nrd1499 15340386

[B25] McCulloch W. S. Pitts W. (1943). A logical calculus of the ideas immanent in nervous activity. Bull. Math. Biophysics 5, 115–133. 10.1007/BF02478259 2185863

[B26] Mulligan G. Mitsiades C. Bryant B. Zhan F. Chng W. J. Roels S. (2007). Gene expression profiling and correlation with outcome in clinical trials of the proteasome inhibitor bortezomib. Blood 109 (8), 3177–3188. 10.1182/blood-2006-09-044974 17185464

[B27] Muzika F. Górecki J. (2022). Identification of the best medium for experiments on chemical computation with Belousov-Zhabotinsky reaction and ferroin-loaded Dowex beads. Reac Kinet. Mech. Cat. 135, 1187–1209. 10.1007/s11144-022-02171-4

[B28] Proskurkin I. S. Smelov P. S. Vanag V. K. (2020). Experimental verification of an opto-chemical "neurocomputer". Phys. Chem. Chem. Phys. 22 (34), 19359–19367. 10.1039/d0cp01858a 32822448

[B29] Rovinskii A. B. Zhabotinskii A. M. Irving R. (1984). Mechanism and mathematical model of the oscillating bromate-ferroin-bromomalonic acid reaction. J. Phys. Chem. 88, 6081–6084. 10.1021/j150669a001

[B30] Steinbock O. Tóth Á. Showalter K. (1995). Navigating Complex Labyrinths: Optimal Paths from Chemical Waves. Science 267, 868–871. 10.1126/science.267.5199.868 17813917

[B31] Sutthiopad M. Luengviriya J. Porjai P. Tomapatanaget B. Müller S. C. Luengviriya C. (2014). Unpinning of spiral waves by electrical forcing in excitable chemical media. Phys. Rev. E 89 (5), 052902. 10.1103/PhysRevE.89.052902 25353856

[B32] Szymanski J. Gorecka J. N. Igarashi Y. Gizynski K. Gorecki J. Zauner K. P. (2011). Droplets with information processing ability. Int. J. Unconv. Comput. 7, 185–200.

[B33] Tanaka M. Nagahara H. Kitahata H. Krinsky V. Agladze K. Yoshikawa K. (2007). Survival versus collapse: abrupt drop of excitability kills the traveling pulse, while gradual change results in adaptation. Phys. Rev. E 76 (1 Pt 2), 016205. 10.1103/PhysRevE.76.016205 17677541

[B34] Tóth Á. Showalter K. (1995). Logic gates in excitable media. J. Chem. Phys. 103, 2058–2066. 10.1063/1.469732

[B35] Waldrop M. M. (2016). The chips are down for Moore's law. Nature 530 (7589), 144–147. 10.1038/530144a 26863965

[B36] Yoshikawa K. Motoike T. I. Yamaguchi T. Igarashi Y. Gorecki J. Gorecka J. N. (2009). Basic information processing operations with pulses of excitation in a reaction-diffusion system. Int. J. Unconv. Comput. 5, 3–37.

[B37] Zhabotinsky A. M. (1964). Periodic liquid phase reactions. Proc. Acad. Sci. USSR 157, 392–395.

[B38] Zhan F. Huang Y. Colla S. Stewart J. P. Hanamura I. Gupta S. (2006). The molecular classification of multiple myeloma. Blood 108, 2020–2028. 10.1182/blood-2005-11-013458 16728703 PMC1895543

